# The Effect of Fe_2_O_3_ Modification on the CeO_2_-MnO_2_/TiO_2_ Catalyst for Selective Catalytic Reduction of NO with NH_3_

**DOI:** 10.3390/molecules30214260

**Published:** 2025-10-31

**Authors:** Yuming Yang, Xue Bian, Jiaqi Li, Zhongshuai Jia, Yuting Bai

**Affiliations:** 1 Baogang Group Mining Research Institute, Baotou 014030, China; yangyuming98@163.com (Y.Y.); 17824833520@163.com (J.L.); jzsoooo@163.com (Z.J.); 2 Inner Mongolia Key Laboratory of Mining and Metallurgical Solid Waste Resource and Green Comprehensive Utilization, Baotou 014030, China; 3 School of Metallurgy, Northeastern University, Shenyang 110819, China; 4 School of Metallurgy and Materials Engineering, Liaoning Institute of Science and Technology, Benxi 117004, China; baiyuting0220@163.com

**Keywords:** NH_3_-SCR, NO_x_, catalyst

## Abstract

High denitration efficiency and strong adaptability to flue gas temperature fluctuations are the core properties of the NH_3_-SCR catalyst. In this study, Fe_2_O_3_ modification is used as a means to explore the mechanism of adding Fe_2_O_3_ to broaden the temperature range of the 6CeO_2_-40MnO_2_/TiO_2_ catalyst during the preparation process. The results show that the 6Fe_2_O_3_-6CeO_2_-40MnO_2_/TiO_2_ catalyst exhibits excellent denitration performance, with a denitration efficiency higher than 90%. The temperature range is from 129 to 390 °C. N_2_ selectivity and resistance to SO_2_ and H_2_O are good, and the denitration performance is significantly improved. When the Fe_2_O_3_ content is 6%, it promotes lattice shrinkage of TiO_2_, improves its dispersion, refines the grain size, and increases the specific surface area of the catalyst. At the same time, Fe_2_O_3_ enhances the chemical adsorption of oxygen on the catalyst surface and increases the proportion of low-cost metal ions, thereby promoting electron transfer between active elements, generating more surface reactive oxygen species, increasing the oxygen vacancy content and adsorption sites for NO_x_ and NH_3_, and significantly improving the redox performance of the catalyst. This effect is particularly conducive to the formation of strong acid sites on the catalyst surface. The NH_3_-SCR reaction on the surface of the 6Fe_2_O_3_-6CeO_2_-40MnO_2_/TiO_2_ catalyst follows both the L-H and E-R mechanisms, with the L-H mechanism being dominant.

## 1. Introduction

Nitrogen oxide (NO_x_) pollution has become one of the main sources of air pollution. Among industrial emissions, those from the power industry account for the largest proportion [1,2]. Excessive NO_x_ in the atmosphere can lead to environmental problems such as acid rain, photochemical smog, eutrophication of water bodies, and the greenhouse effect [3]. The country’s regulations on industrial NO_x_ emissions are becoming increasingly stringent, so environmental protection in metallurgy, building materials, glass, and other industries is under great pressure. The selective catalytic reduction (SCR) method is currently the most widely used flue gas denitration technology for controlling NO_x_ emissions from industrial sources [4]. A more mature catalyst for this method is the V_2_O_5_-WO_3_(MoO_3_)/TiO_2_ catalyst, which belongs to the category of medium-temperature catalysts [5]. To avoid sulfur dioxide poisoning, it is placed in the flue gas treatment process after desulfurization and dust removal. Therefore, the flue gas needs to be reheated before denitration, which imposes greater economic pressure on enterprises. Moreover, the vanadium component in the catalyst is toxic, and the recycling and disposal of waste catalysts can easily cause pollution. The application prospects of non-toxic SCR catalysts capable of denitrating at low temperatures are very promising.

Among the many transition metal oxides, MnO_x_ has the advantages of multiple valence states, strong acidity, and low cost, while CeO_2_ has excellent oxygen storage and release capacity as well as redox performance. Thus, low-temperature SCR denitration catalysts with MnO_x_ and CeO_2_ as the main active components or additives have become a hot topic in the field of denitration catalysts [6,7]. Liu et al. [8] used the sol–gel method to prepare a series of Mn-Ti oxide catalysts. Through experiments, they found that these catalysts have high low-temperature SCR activity. The excellent low-temperature NH_3_-SCR activity can be attributed to appropriate structural properties, amorphous manganese oxides with equal proportions of Mn^3+^/Mn^4+^, good low-temperature reducibility, and rich surface acid sites. Liu et al. [9] studied the performance of the Mn-Ce-Ti mixed oxide catalyst prepared by the hydrothermal method for the selective catalytic reduction of NO_x_ by NH_3_ in the presence of oxygen. The results show that the Mn-Ce-Ti catalyst has excellent NH_3_-SCR activity, strong resistance to H_2_O and SO_2_, and a wide operating temperature window. On the basis of catalyst characterization, the double redox cycles (Mn^4+^ + Ce^3+^, Mn^4+^ + Ti^3+^ ↔ Mn^3+^ + Ce^4+^, Mn^4+^ + Ti^3+^) and the amorphous structure play key roles in the catalytic properties for NO_x_ reduction.

China is one of the countries with the richest iron ore resources in the world. If Fe_2_O_3_ is used in combination with other metal oxides for the research and development of denitration catalysts, it can realize the efficient denitration of industrial source flue gas at low cost, which is a catalyst preparation technology route that aligns with China’s national conditions [10]. Many studies have shown that Fe_2_O_3_ modification can improve the denitration performance and anti-toxicity performance of SCR catalysts. Shi et al. [11] investigated the effects of iron in the Mn/Ti catalyst system on NH_3_-SCR activity and the water-induced inactivation. The results show that the addition of iron promotes the dispersion of surface species, improves the valence state of manganese, and enhances the low-temperature activity. Chen et al. [12] prepared a new type of Fe-Mn mixed oxide catalyst, of which the Fe(0.4) MnO_x_ catalyst has the highest catalytic activity. At 120 °C and the airspeed of 30,000 h^−1^, the NO_x_ conversion rate is 98.8%, and the N_2_ selectivity is 100%. The characterization results show that there is a strong interaction between iron oxide and manganese oxide, which leads to the formation of the Fe_3_Mn_3_O_8_ phase in the catalyst. The electron transfer between Fe^n+^ and Mn^n+^ ions may be the reason for the long lifespan of the Fe(0.4) MnO_x_ catalyst. Mu et al. [13] prepared the Fe_2_O_3_-MnO_2_/TiO_2_ catalyst using an ethylene glycol-assisted impregnation method. The catalyst exhibited excellent low-temperature activity, a low apparent activation energy, and strong resistance to sulfur poisoning. The characterization results indicate that the catalyst has better dispersion, smaller particles, and that Fe is partially doped into the TiO_2_ lattice, forming a Fe-O-Ti structure. This enhances the electronic induction effect and increases the proportion of surface chemisorbed oxygen. NO oxidation is enhanced through the “fast SCR” process, which is beneficial for low-temperature SCR activity. Zhao et al. [14] studied the low-temperature denitration properties of catalysts with different Fe contents. The results show that Fe_4_Mn_7_Ce_3_, doped with 4 wt% Fe on the basis of Mn_7_Ce_3_, has the best DeNO_x_ performance. The doping of Fe makes the oxides evenly distributed on the catalyst surface, promotes the valence state cycle of the catalyst, generates more Mn^4+^ and Ce^3+^, and shifts the optimal temperature of DeNO_x_ toward lower temperatures. At the same time, the Lewis acid sites on the catalyst surface are enhanced, which promotes the formation of amides and facilitates the DeNO_x_ reaction. Qiu et al. [15] prepared a Fe-modified Mn-Co-Ce/TiO_2_-SiO_2_ catalyst by the impregnation and sulfuric acid methods and evaluated its low-temperature NH_3_-SCR performance in the presence of SO_2_ and H_2_O. The study found that adding Fe to MnCoCe/Ti-Si plays an important role in enhancing the anti-sulfur and anti-water poisoning properties of MnCoCe/Ti-Si. When the optimal MnFeCoCe/Ti-Si catalyst is operated at a reaction temperature of 160 °C, an SO_2_ concentration of 50 ppm, and an H_2_O concentration of 100%, the NO_x_ conversion rate is as high as 93%. The interaction between MnO_x_ and FeO_x_ improves the sulfur and water resistance more effectively compared to other bimetallic interactions.

Through previous research and early experimental demonstrations by this group, it is concluded that when MnO_2_ and CeO_2_ account for 0.4 and 0.06 of the overall mass of the catalyst, respectively, the denitration performance of the CeO_2_-MnO_2_/TiO_2_ catalyst is relatively superior [16]. When Fe_2_O_3_ is used as an active component or additive, it can improve the anti-poisoning and anti-sintering capabilities of the catalyst [17]. Meanwhile, the interaction among Fe, Mn, and Ce is helpful in increasing the number of active sites of the catalyst and improving the dispersion state of the active components on the support [18,19]. In order to clarify the mechanism by which Fe_2_O_3_ modification’s influence on the denitration performance of the 6CeO_2_-40MnO_2_/TiO_2_ catalyst, this study employs XRD, BET, XPS, SEM, TEM, H_2_-TPR, NH_3_-TPD, and in situ DRIFTS to investigate the effects of Fe_2_O_3_ on denitration performance, catalytic material morphology, surface acidity, redox capacity, and the catalytic mechanism during the modification of the 6CeO_2_-40MnO_2_/TiO_2_ catalyst. This study aims to provide theoretical and technical support for the research, development, and application of rare earth-modified denitration catalysts.

## 2. Results and Discussion

### 2.1. Morphology and Structure of the Catalysts

#### 2.1.1. XRD, BET Surface Area and Pore Morphology

**Figure 1:**
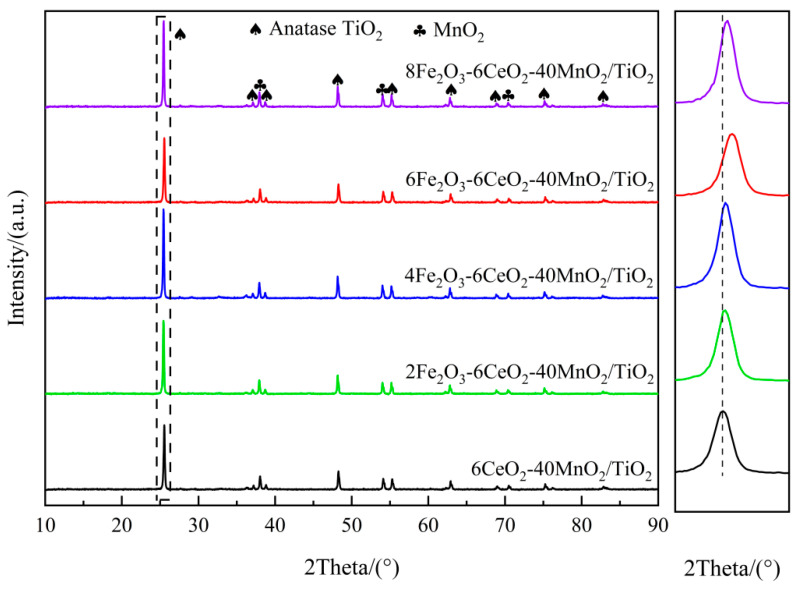
XRD patterns of xFe_2_O_3_-6CeO_2_-40MnO_2_/TiO_2_ catalysts.

Figure 1 shows the XRD spectrum of the xFe_2_O_3_-6CeO_2_-40MnO_2_/TiO_2_ catalyst. It can be seen that there are obvious anatase-type TiO_2_ and MnO_2_ diffraction peaks in all catalysts, but no obvious Fe_2_O_3_ and CeO_2_ diffraction peaks appear, indicating that they exist in an amorphous state or are highly dispersed on the surface of the TiO_2_ support. When the Fe_2_O_3_ content increases, the TiO_2_ diffraction peak at 25.3° shifts gradually to higher angles. Combined with the Bragg equation (2dsinθ= nλ), it can be observed that the diffraction angle is inversely proportional to the lattice spacing. Therefore, after the Fe_2_O_3_ content increases, the lattice spacing and grain size of TiO_2_ in the catalyst decrease, and the lattice spacing of TiO_2_ in the 6Fe_2_O_3_-6CeO_2_-40MnO_2_/TiO_2_ catalyst decreases the most, indicating that adding an appropriate amount of Fe_2_O_3_ can promote the lattice shrinkage of TiO_2_. This point is also confirmed by the TEM test results presented later. This shrinkage and the resulting decrease in crystallinity increase the overall specific surface area of the catalyst, thereby enhancing the exposure area of the active component and improving the denitration performance of the catalyst.

The specific surface area and pore structure characteristics of the xFe_2_O_3_-6CeO_2_-40MnO_2_/TiO_2_ catalyst were analyzed using the N_2_ adsorption–desorption measurement method, and the results are presented in Table 1.

Studies have shown that increasing the specific surface area is conducive to improving catalytic performance, and micropores and mesopores can provide a larger inner surface area and pore volume in the SCR reaction [20]. As depicted in Table 1, when the Fe_2_O_3_ content increases, the specific surface area and pore capacity of the catalyst increase, while the pore size decreases. The specific surface area and pore capacity of the 6Fe_2_O_3_-6CeO_2_-40MnO_2_/TiO_2_ catalyst increase to 87.73 m^2^·g^−1^ and 0.268 cm^3^·g^−1^, respectively, and the average pore size decreases to 12.22 nm. Combined with XRD analysis, the increase in Fe_2_O_3_ content can inhibit the crystallization and crystal growth of TiO_2_, improve the overall dispersion of the catalyst, and increase the specific surface area. When the Fe_2_O_3_ content continues to increase to 8%, the specific surface area (87.75 m^2^·g^−1^) and pore capacity (0.254 cm^3^·g^−1^) of the catalyst remain nearly constant.

#### 2.1.2. Morphological Analysis

**Figure 2:**
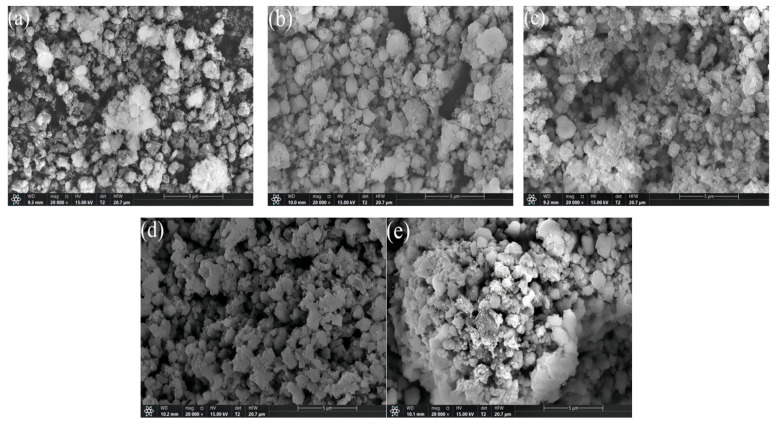
SEM images of xFe_2_O_3_-6CeO_2_-40MnO_2_/TiO_2_ catalysts. ((**a**) 6CeO_2_-40MnO_2_/TiO_2_; (**b**) 2Fe_2_O_3_-6CeO_2_-40MnO_2_/TiO_2_; (**c**) 4Fe_2_O_3_-6CeO_2_-40MnO_2_/TiO_2_; (**d**) 6Fe_2_O_3_-6CeO_2_-40MnO_2_/TiO_2_; (**e**) 8Fe_2_O_3_-6CeO_2_-40MnO_2_/TiO_2_).

The SEM image of the xFe_2_O_3_-6CeO_2_-40MnO_2_/TiO_2_ catalyst is shown in Figure 2. It can be observed that the catalyst is mainly composed of particles with uneven sizes, and there are varying degrees of agglomeration. As illustrated in Figure 2a, large and dense aggregates can be clearly observed on the surface of the 6CeO_2_-40MnO_2_/TiO_2_ catalyst. The active components are encapsulated within these aggregates and cannot participate in the denitration reaction, which negatively affects the catalyst’s denitration performance. With the increase in Fe_2_O_3_ content, the surface particles of the catalyst become gradually finer, and the agglomeration and encapsulation phenomena decrease, indicating a relatively better pore structure. In Figure 2d, the surface particles of the 6Fe_2_O_3_-6CeO_2_-40MnO_2_/TiO_2_ catalyst exhibit similar sizes and a uniform distribution. A good pore structure with more micropores can provide a larger contact area for flue gas, thereby facilitating a more thorough removal of NO_x_ during the denitration reaction.

**Figure 3:**
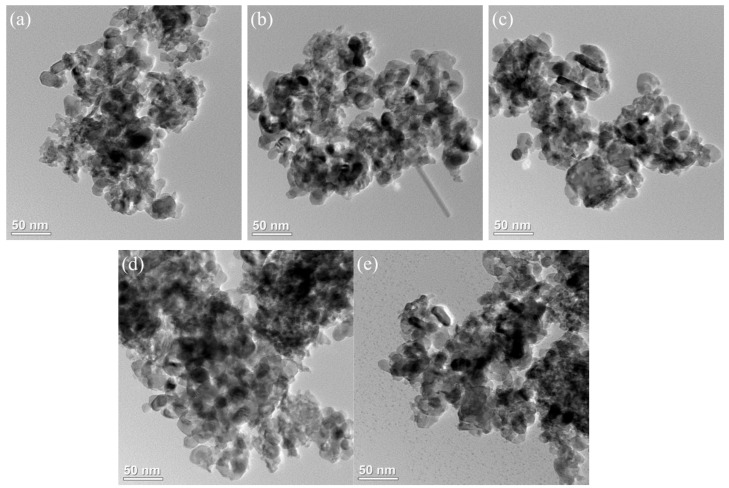
TEM images of xFe_2_O_3_-6CeO_2_-40MnO_2_/TiO_2_ catalysts.((**a**) 6CeO_2_-40MnO_2_/TiO_2_; (**b**) 2Fe_2_O_3_-6CeO_2_-40MnO_2_/TiO_2_; (**c**) 4Fe_2_O_3_-6CeO_2_-40MnO_2_/TiO_2_; (**d**) 6Fe_2_O_3_-6CeO_2_-40MnO_2_/TiO_2_; (**e**) 8Fe_2_O_3_-6CeO_2_-40MnO_2_/TiO_2_).

The TEM image of the xFe_2_O_3_-6CeO_2_-40MnO_2_/TiO_2_ catalyst is shown in Figure 3. Most of the catalyst is composed of particles of different sizes, and the dark-colored small particles are generally adsorbed on the surface of the large particles (the TiO_2_ support) in a loaded state. Figure 3a shows that when the Fe_2_O_3_ content is low, the dispersion of the particles on the catalyst surface is poor, and there is a significant agglomeration phenomenon, which is consistent with the results from the SEM image. As the Fe_2_O_3_ content increases, the number of active substance particles in the figure gradually increases and becomes more dispersed. In Figure 3d, the surface of the 6Fe_2_O_3_-6CeO_2_-40MnO_2_/TiO_2_ catalyst has the largest number of active substances exposed and is the most evenly distributed, which is conducive to the acid cycle and redox cycle of the NH_3_-SCR reaction. When the Fe_2_O_3_ content continues to increase, the number of active substance particles does not change significantly, but the agglomeration phenomenon becomes more pronounced.

**Figure 4:**
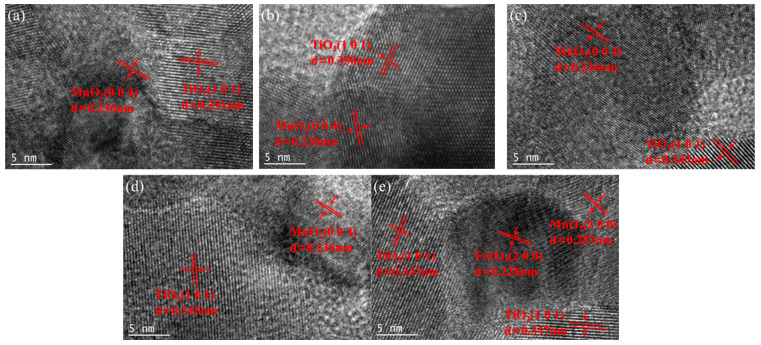
Lattice fringe pattern of xFe_2_O_3_-6CeO_2_-40MnO_2_/TiO_2_ catalysts ((**a**) 6CeO_2_-40MnO_2_/TiO_2_; (**b**) 2Fe_2_O_3_-6CeO_2_-40MnO_2_/TiO_2_; (**c**) 4Fe_2_O_3_-6CeO_2_-40MnO_2_/TiO_2_; (**d**) 6Fe_2_O_3_-6CeO_2_-40MnO_2_/TiO_2_; (**e**) 8Fe_2_O_3_-6CeO_2_-40MnO_2_/TiO_2_).

Figure 4 presents a high-resolution lattice stripe image of the xFe_2_O_3_-6CeO_2_-40MnO_2_/TiO_2_ catalyst. The lattice stripe spacing of *d* = 0.351 nm corresponds to the TiO_2_(101) crystal surface, while the lattice stripe spacings of *d* = 0.236 nm and *d* = 0.287 nm correspond to the MnO_2_(004) and MnO_2_(200) crystal surfaces, respectively. The lattice stripe spacing of *d* = 0.228 nm corresponds to the Fe_2_O_3_(200) crystal surface. The figure shows that when the Fe_2_O_3_ content varies, the lattice stripe spacings corresponding to the MnO_2_(004) and MnO_2_(200) crystal surfaces remain almost unchanged. The lattice stripe spacing corresponding to the TiO_2_(101) crystal surface decreases with increasing Fe_2_O_3_ content, indicating that increasing the amount of Fe_2_O_3_ added can promote lattice shrinkage of TiO_2_ and increase the specific surface area of the catalyst, which is consistent with the results of XRD and BET analyses. When the Fe_2_O_3_ content increased to 8%, the Fe_2_O_3_(200) crystal surface appeared in Figure 4e, indicating that some Fe_2_O_3_ was present as dispersed particles on the surface of the TiO_2_ support at this point.

### 2.2. Surface Properties of Catalysts

**Figure 5:**
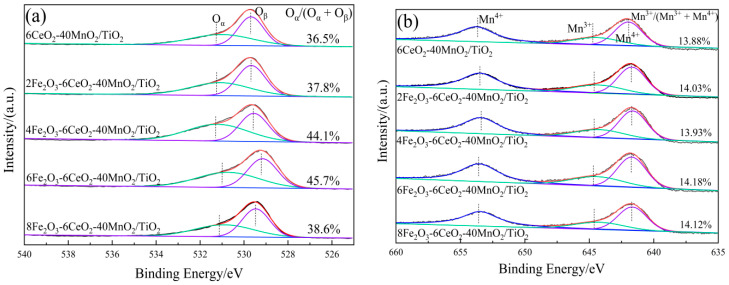

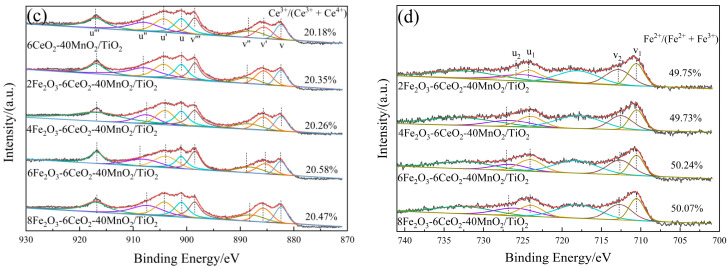
XPS spectra of xFe_2_O_3_-6CeO_2_-40MnO_2_/TiO_2_ catalysts ((**a**) O 1s; (**b**) Mn 2p; (**c**) Ce 3d; (**d**) Fe 2p).

In order to explore the influence of Fe_2_O_3_ addition on the surface element distribution and redox performance of the xFe_2_O_3_-6CeO_2_-40MnO_2_/TiO_2_ catalyst, XPS tests were performed on catalysts with different Fe_2_O_3_ addition amounts. The results are presented in Figure 5 and Table 2.

Figure 5a shows the O 1s spectrum of the xFe_2_O_3_-6CeO_2_-40MnO_2_/TiO_2_ catalyst. Two characteristic peaks appear in the figure at approximately 532.2 eV and 530.1 eV, corresponding to chemically adsorbed oxygen (O_α_) and lattice oxygen (O_β_), respectively. With increasing Fe_2_O_3_ content, the O_α_ peak tends to shift to lower binding energies, and the ratio of O_α_ to the total surface oxygen (O_α_/(O_α_ + O_β_)) first rises and then decreases. Since O_α_ exhibits higher mobility than O_β_, it is generally considered to be more reactive in redox reactions [21]. The xFe_2_O_3_-6CeO_2_-40MnO_2_/TiO_2_ catalyst exhibits a maximum O_α_/(O_α_ + O_β_) value of 45.7%, indicating that an appropriate amount of Fe_2_O_3_ can promote the formation of highly active O_α_ species and more oxygen vacancies on the catalyst surface. This enhances the catalyst’s denitration performance, which is consistent with the denitration performance test results presented in Section 2.6.

The Fe 2p spectrum of the xFe_2_O_3_-6CeO_2_-40MnO_2_/TiO_2_ catalyst is shown in Figure 5d. The Fe 2p spectrum suggests that the Fe elements in the catalyst coexist in the form of Fe^3+^(u_2_,v_2_) and Fe^2+^(u_1_,v_1_). After the Fe_2_O_3_ content is increased, the proportion of Fe^2+^ on the catalyst surface relative to the total Fe elements (Fe^2+^/(Fe^2+^ + Fe^3+^)) also increases, and the Fe^2+^/(Fe^2+^ + Fe^3+^) value of the 6Fe_2_O_3_-6CeO_2_-40MnO_2_/TiO_2_ catalyst is as high as 50.24%, indicating that increasing the Fe_2_O_3_ content can promote the redox reaction between Fe^3+^ and Fe^2+^: Fe^3+^ ↔ Fe^2+^ + Cat-[O]. This provides more active sites and oxygen vacancies, which are conducive to improving the denitration activity of the catalyst [22]. In addition, due to the coexistence of Fe^2+^ and Fe^3+^, there is a Fe^2+^/Fe^3+^ redox couple in the catalyst, which facilitates the storage and release of oxygen on the catalyst surface and enhances the redox capacity of the catalyst.

Figure 5b,c show the Mn 2p and Ce 3d spectra of the xFe_2_O_3_-6CeO_2_-40MnO_2_/TiO_2_ catalyst, respectively. As can be seen from the figures, when the amount of Fe_2_O_3_ added varies, the positions of the characteristic peaks and the peak areas do not change significantly. This indicates that the surface Ce^4+^/Ce^3+^ and Mn^3+^/Mn^4+^ ratios remain relatively constant, suggesting that varying the amount of Fe_2_O_3_ does not significantly alter the valence states of Mn and Ce on the catalyst surface.

### 2.3. Acidic Sites Distribution of the Catalyst

**Figure 6:**
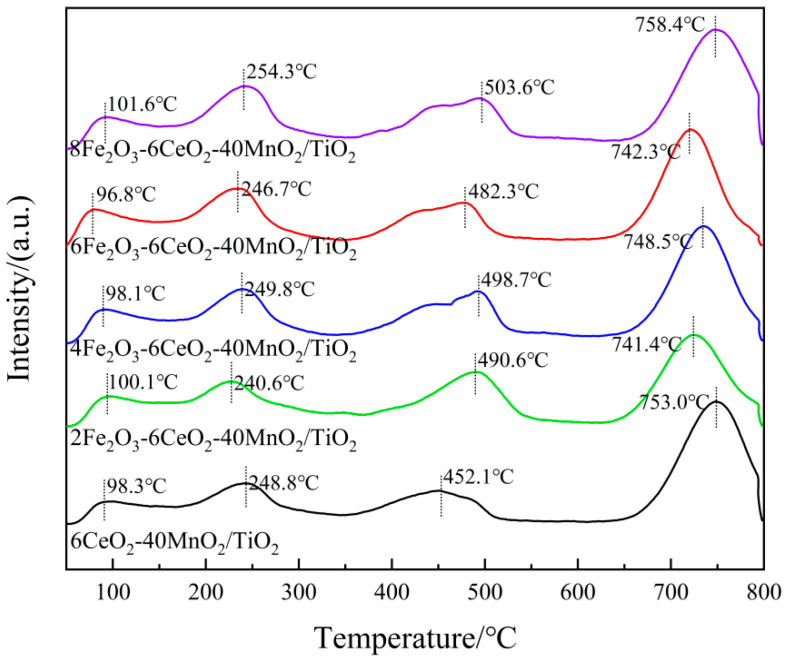
NH_3_-TPD pattern of xFe_2_O_3_-6CeO_2_-40MnO_2_/TiO_2_ catalysts.

In order to study the influence of acid density, acid strength, and acid strength distribution on the catalyst surface on denitration performance, the xFe_2_O_3_-6CeO_2_-40MnO_2_/TiO_2_ catalyst was analyzed using NH_3_-TPD. The results are shown in Figure 6 and Table 3. According to existing studies, the desorption peak located in the range of 25~200 °C represents a weak acid site, which is mainly derived from NH_3_ adsorbed by Lewis acid sites and physically adsorbed NH_3_. The peak between 200 and 400 °C corresponds to medium-strong acid sites, mainly due to NH_3_ adsorbed by Lewis acid sites. In addition, the desorption peak above 400 °C corresponds to strong acid sites, which belong to NH_3_ adsorbed by Brønsted acid sites [23]. As shown in Figure 6, with the change in Fe_2_O_3_ content, the position of the desorption peak of each acid site has not shifted significantly. According to the data in Table 3, with the increase in Fe_2_O_3_ content, the NH_3_ adsorption capacity of weak and medium-strong acid sites has not changed significantly, whereas the adsorption capacity of strong acids has increased. The 6Fe_2_O_3_-6CeO_2_-40MnO_2_/TiO_2_ catalyst exhibits the highest adsorption capacity. This indicates that increasing Fe_2_O_3_ promotes the formation of strong acid sites, thereby increasing the number of Brønsted acid sites on the catalyst surface. This complements the role of CeO_2_ in promoting the formation of weak and medium-strong acid sites (e.g., enhancing Lewis acid sites) [24]. Lewis acid sites are usually associated with the adsorption and activation of NH_3_, and are key active sites for low-temperature SCR; The Brønsted acid sites adsorb NH_3_ to form NH_4_^+^, which can enhance the activity at medium to high temperatures through a “rapid SCR” reaction (NH_4_^+^ + NO_2_ → N_2_ + H_2_O) and affect N_2_ selectivity (reducing excessive oxidation of NH_3_). After doping with Fe_2_O_3_, the number of strong acid sites on the catalyst surface increases, and the number of Brønsted acid sites on the catalyst surface increases. This is the main reason for the significant enhancement of high-temperature catalytic activity and N_2_ selectivity in the 6Fe_2_O_3_-6CeO_2-_40MnO_2_/TiO_2_ catalyst shown in Section 2.5.

### 2.4. Redox Performance of the Catalysts

**Figure 7:**
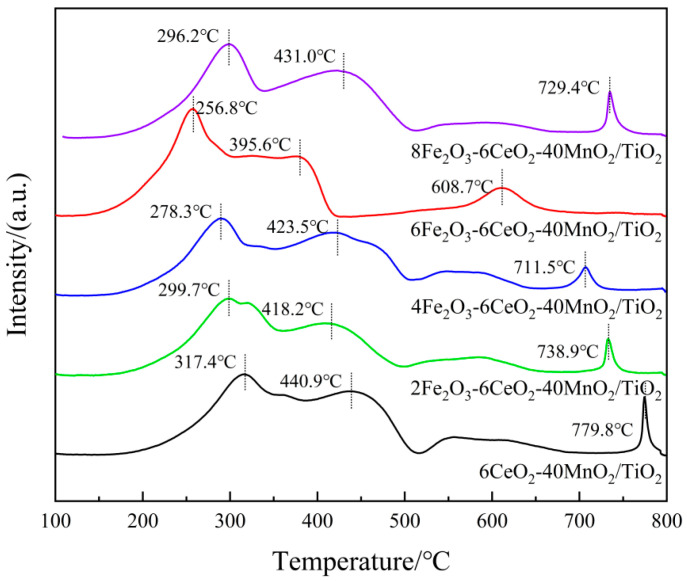
H_2_-TPR pattern of xFe_2_O_3_-6CeO_2_-40MnO_2_/TiO_2_ catalysts.

In the NH_3_-SCR reaction process, the redox performance of the catalyst plays a key role in the cyclic operation of the denitration reaction. To study the influence of Fe_2_O_3_ addition on the redox characteristics of the catalyst, the xFe_2_O_3_-6CeO_2_-40MnO_2_/TiO_2_ catalysts with different Fe_2_O_3_ contents were characterized and analyzed by H_2_-TPR. The results are shown in Figure 7 and Table 4.

In Figure 7, each catalyst sample demonstrates three reduction peaks. Among these, the reduction peaks ranging from 250 to 350 °C correspond to the reduction of Fe^3+^ → Fe^2+^, those ranging from 350 to 520 °C correspond to the reduction of Mn^4+^ → Mn^3+^, and those ranging from 520 to 800 °C correspond to the reduction of Ce^4+^ → Ce^3+^ [25,26,27]. When the amount of Fe_2_O_3_ added was gradually increased to 6%, the positions of each reduction peak shifted toward lower temperatures, suggesting that an appropriate increase in Fe_2_O_3_ content can enhance the activity of oxygen species and improve the reduction capacity of the catalyst. When the Fe_2_O_3_ content continued to increase to 8%, the reduction peaks shifted toward higher temperatures again. This may be because the addition of a small amount of Fe_2_O_3_ enhances the interaction between Fe_2_O_3_, MnO_2_, and CeO_2_ (e.g., Fe^3+^ + Mn^3+^ ↔ Fe^2+^ + Mn^4+^, Fe^3+^ + Ce^3+^ ↔ Fe^2+^ + Ce^4+^). However, excessive Fe_2_O_3_ content leads to the disruption of the electron transfer balance among the active components, thus decreasing the reduction capacity of the catalyst. In addition, as the Fe_2_O_3_ content increases, the H_2_ consumption associated with Fe^3+^ → Fe^2+^ gradually increases. When the H_2_ consumption of the catalyst sample increases, the corresponding denitrification activity of the sample also improves. At the same time, the H_2_ consumption for Mn^4+^ → Mn^3+^ and Ce^4+^ → Ce^3+^ also increases, demonstrating that increasing the amount of Fe_2_O_3_ can increase the number of active species on the catalyst surface, thereby increasing the relative content of surface chemisorbed oxygen, which is beneficial for the NH_3_-SCR reaction. This observation is consistent with the XPS analysis results.

### 2.5. Catalytic Activity of xFe_2_O_3_-6CeO_2_-40MnO_2_/TiO_2_ Catalyst

#### 2.5.1. The Denitration Performance of xFe_2_O_3_-6CeO_2_-40MnO_2_/TiO_2_ Catalyst

**Figure 8:**
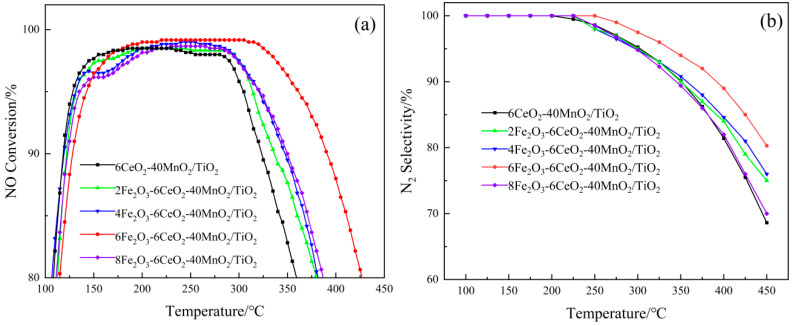
The denitration performance of xFe_2_O_3_-6CeO_2_-40MnO_2_/TiO_2_ catalysts. (**a**) NO Conversion; (**b**) N_2_ Selectivity).

Figure 8 shows the experimental results of the xFe_2_O_3_-6CeO_2_-40MnO_2_/TiO_2_ catalyst in terms of denitration performance. As shown in Figure 8a, without the addition of Fe_2_O_3_, the denitration effect of the 6CeO_2_-40MnO_2_/TiO_2_ catalyst is the least ideal. The denitration efficiency is above 80% within the temperature range of 110~360 °C, while the temperature range where the efficiency exceeds 90% is 118~321 °C. With the gradual increase in Fe_2_O_3_ content, the denitration performance of the catalyst also shows an increasing trend. When the Fe_2_O_3_ content reaches 6%, the catalyst exhibits optimal denitration performance. At this point, the temperature range where the denitration efficiency exceeds 80% extends to 115~425 °C, and the range where the efficiency is higher than 90% is 129~390 °C. Between 220 and 305 °C, the denitration efficiency reaches its peak at 99.17%. However, when the Fe_2_O_3_ content is further increased to 8%, the denitration performance of the catalyst is weakened.

As shown in Figure 8b, the xFe_2_O_3_-6CeO_2_-40MnO_2_/TiO_2_ catalyst exhibits 100% N_2_ selectivity at temperatures below 200 °C. Nevertheless, when the temperature rises above 200 °C, the selectivity of N_2_ begins to decrease, indicating that the NH_3_ adsorbed on the catalyst surface undergoes excessive oxidation at higher temperatures, resulting in the generation of the by-product N_2_O. With the increase in Fe_2_O_3_ content, the downward trend of N_2_ selectivity gradually slows down. Among them, the 6Fe_2_O_3_-6CeO_2_-40MnO_2_/TiO_2_ catalyst exhibits optimal N_2_ selectivity, which can still be maintained at more than 80% at 450 °C, with the slowest decline rate. However, when the Fe_2_O_3_ content is further increased to 8Fe_2_O_3_-6CeO_2_-40MnO_2_/TiO_2_, the N_2_ selectivity of the catalyst deteriorates.

#### 2.5.2. The Anti-SO_2_ and Anti-H_2_O Properties of xFe_2_O_3_-6CeO_2_-40MnO_2_/TiO_2_ Catalyst

**Figure 9:**
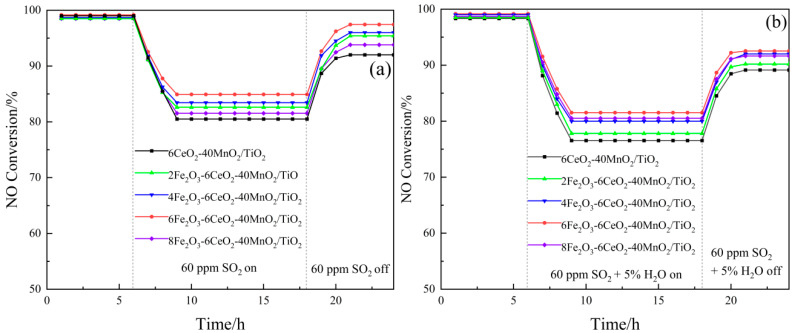
SO_2_ and H_2_O resistance of xFe_2_O_3_-6CeO_2_-40MnO_2_/TiO_2_ catalysts ((**a**) SO_2_; (**b**) SO_2_ and H_2_O).

The test results of the SO_2_ and H_2_O resistance of the xFe_2_O_3_-6CeO_2_-40MnO_2_/TiO_2_ catalyst are shown in Figure 9. Each group of catalysts was first stabilized at 200 °C for 6 h, and then 60 ppm SO_2_ was introduced into the simulated flue gas. The denitration efficiency of the catalysts with SO_2_ contents of 0%, 2%, 4%, 6%, and 8% decreased to 80.5%, 82.6%, 83.4%, 84.8%, and 81.5%, respectively, and then gradually stabilized. After 18 h, the introduction of SO_2_ was stopped, and the denitration efficiency gradually recovered to a certain value and remained stable. Among them, the denitration performance of the 6CeO_2_-40MnO_2_/TiO_2_ catalyst was most affected by SO_2_, while the 6Fe_2_O_3_-6CeO_2_-40MnO_2_/TiO_2_ catalyst was least affected. Its denitration efficiency rose to a higher value after the introduction of SO_2_ was stopped. This is probably due to the fact that an appropriate amount of Fe_2_O_3_ can increase the dispersion of the active substance on the catalyst surface, providing more active sites, thereby improving the anti-SO_2_ performance of the catalyst.

Also after constant temperature denitration at 200 °C for 6 h, 60 ppm SO_2_ and water vapor with a volume fraction of 5% were simultaneously introduced into the mixed flue gas, and the denitration efficiency of the catalyst with FeO_2_ content of 0%, 2%, 4%, 6%, and 8% was reduced to 76.5%, 77.8%, 80.1%, 81.6%, and 80.5%, respectively. After that, it gradually stabilized, and the introduction of SO_2_ and H_2_O was stopped at 18 h. Afterward, the denitration efficiency recovered to some extent. Among them, the 6CeO_2_-40MnO_2_/TiO_2_ catalyst was most affected by SO_2_ and H_2_O, while the 6Fe_2_O_3_-6CeO_2_-40MnO_2_/TiO_2_ catalyst exhibited good anti-SO_2_ and anti-H_2_O properties.

#### 2.5.3. Stability of Catalytic Activity of 6Fe_2_O_3_-6CeO_2_-40MnO_2_/TiO_2_ Catalyst

**Figure 10:**
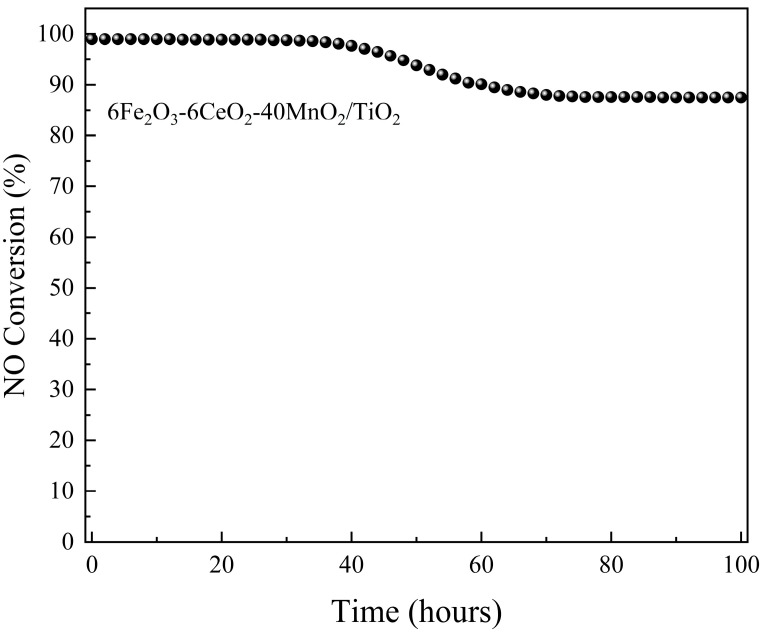
Stability of catalytic activity of 6Fe_2_O_3_-6CeO_2_-40MnO_2_/TiO_2_ catalyst.

To evaluate the stability of the catalytic activity of the 6Fe_2_O_3_-6CeO_2_-40MnO_2_/TiO_2_ catalyst, its performance was continuously monitored for 100 h at 240 °C. Denitrification efficiency was recorded at two-hour intervals. The results are presented in Figure 10. For the first 34 h, the catalytic activity remained stable at about 99%. From 36 h onward, a decline in denitrification efficiency was observed, reaching 87.5% by 76 h. This level was maintained until the conclusion of the test.

#### 2.5.4. Kinetic Analysis of xFe_2_O_3_-6CeO_2_-40MnO_2_/TiO_2_ Catalyst

In order to explore the effect of Fe_2_O_3_ modification on the reaction rate of the CeO_2_-MnO_2_/TiO_2_ catalyst, the kinetic characteristics of its denitration reaction were analyzed in this study. By comparing the apparent activation energy of the catalytic reaction, different reaction paths can be identified, and the adsorption and desorption behavior of the reactants on the catalyst surface can be further understood [28]. The reaction rate constant *k* is calculated based on Equation (3).

(1) $$k=-\frac{V}{W}\ln(1-X)$$

where *k* is the reaction rate constant in cm^3^·g^−1^·s^−1^, *V* represents the gas flow rate (cm^3^·s^−1^), *W* is the mass of the catalyst (g), and *X* represents the conversion rate of NO. After calculating the *k* value using Equation (3) and combining it with the specific values of *R* and *T*, the three-point fitting method is applied to the Arrhenius plot. The expression is as follows (Equation (4)):

(2) lnk = lnA−Ea/(RT)

where *A* is the precursor (cm^3^·g^−1^·s^−1^), *Ea* represents the apparent activation energy (J·mol^−1^), *R* is the universal gas constant, which has a value of 8.314 J·K^−1^·mol^−1^, and *T* is the reaction temperature (K). A graph is drawn with 1/*T* as the abscissa and the natural logarithm of the reaction rate *k* is plotted as the ordinate. There is a linear relationship between 1/T and ln*k*, and the reaction activation energy *Ea* can be obtained after linear regression.

**Figure 11:**
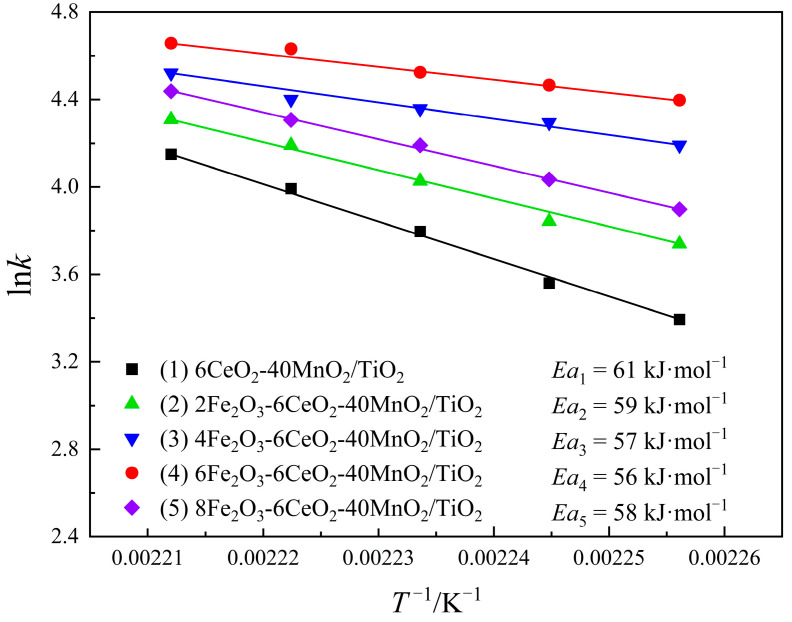
Kinetic diagrams of xFe_2_O_3_-6CeO_2_-40MnO_2_/TiO_2_ catalysts.

Combined with the test results of the denitration performance of the catalyst, the denitration reaction dynamics of the xFe_2_O_3_-6CeO_2_-40MnO_2_/TiO_2_ catalyst were studied using Equations (3) and (4). The results are shown in Figure 11. As can be obtained from Equation (4), ln*k* has a linear relationship with *T*^−1^. The smaller the absolute value of the slope, the smaller the reaction activation energy *Ea*, and the easier the denitration reaction proceeds. As the Fe_2_O_3_ content increases, the activation energy of the catalyst gradually decreases. The 6Fe_2_O_3_-6CeO_2_-40MnO_2_/TiO_2_ catalyst has a minimum activation energy of 56 kJ·mol^−1^. In addition, as the reaction temperature increases, the reaction rate of the catalyst increases. At the same temperature, the reaction rates follow the order: 6Fe_2_O_3_-6CeO_2_-40MnO_2_/TiO_2_ > 4Fe_2_O_3_-6CeO_2_-40MnO_2_/TiO_2_ > 8Fe_2_O_3_-6CeO_2_-40MnO_2_/TiO_2_ > 2Fe_2_O_3_-6CeO_2_-40MnO_2_/TiO_2_ > 6CeO_2_-40MnO_2_/TiO_2_, which is consistent with the denitration performance test results shown in Figure 10.

### 2.6. Catalytic Mechanism Analysis of xFe_2_O_3_-6CeO_2_-40MnO_2_/TiO_2_ Catalyst

Through the above analysis, the catalytic activity of the xFe_2_O_3_-6CeO_2_-40MnO_2_/TiO_2_ catalyst is influenced by a variety of factors, and one of the main reasons is the variation in acidic sites. The SCR reaction is a heterogeneous gas–solid reaction catalyzed by a solid catalyst, which requires the catalyst to have sufficient active sites to adsorb the reaction gases. In order to study the changes in the adsorption characteristics of the 6Fe_2_O_3_-6CeO_2_-40MnO_2_/TiO_2_ catalyst during the low-temperature NH_3_-SCR reaction and to explore the catalytic reaction mechanism on the catalyst surface, in this part, in situ DRIFTS was used to investigate the interaction between the reaction gases (NH_3_ and NO) and the catalyst surface.

(1) Adsorption of NH_3_

**Figure 12:**
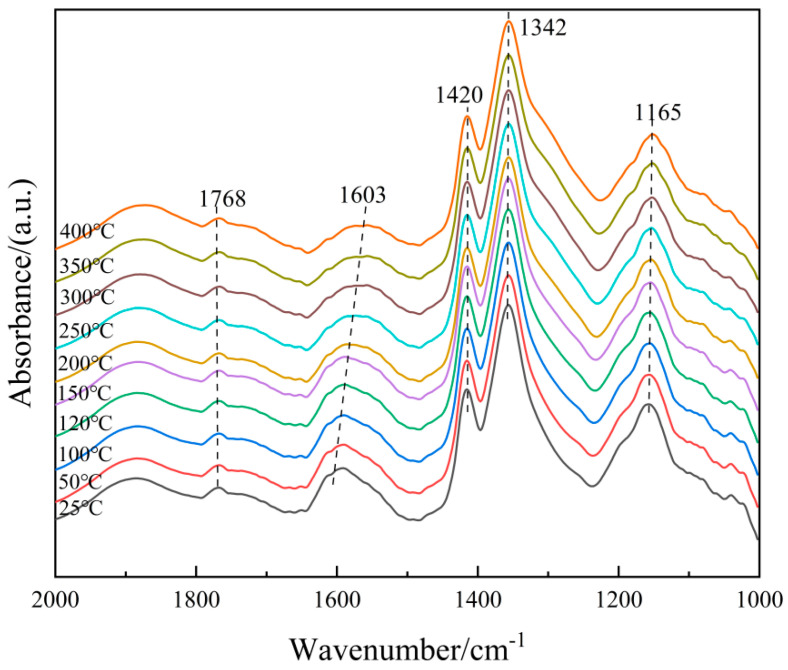
In situ DRIFTS spectra of NH_3_ adsorption on 6Fe_2_O_3_-6CeO_2_-40MnO_2_/TiO_2_ catalyst.

In order to further clarify the properties of the NH_3_(ad) species on the surface of the 6Fe_2_O_3_-6CeO_2_-40MnO_2_/TiO_2_ catalyst, in situ DRIFTS of NH_3_ adsorption at different temperatures were conducted, and the results are shown in Figure 12. Based on the spectrum of the 6Fe_2_O_3_-6CeO_2_-40MnO_2_/TiO_2_ catalyst, the bands that appear at 1768 cm^−1^, 1603 cm^−1^, 1342 cm^−1^, and 1141 cm^−1^ correspond to NH_3_ molecules coordinated to Lewis acid sites, while the band at 1420 cm^−1^ corresponds to NH_4_^+^ ions adsorbed at Brønsted acid sites [29,30]. As the temperature increases, the weakening of peak intensity is mainly due to thermal desorption and possible decomposition, and the attenuation rate is related to the adsorption enthalpy. By comparison, the adsorption peaks corresponding to Brønsted acid sites weaken more rapidly, probably indicating that Lewis acid sites have stronger thermal stability due to higher adsorption enthalpy. Therefore, at low temperatures, NH_3_ adsorption in the catalytic reaction involves both Lewis and Brønsted acid sites, whereas at high temperatures, NH_3_ adsorption mainly occurs at Lewis acid sites.

(2) Co-adsorption of NO + O_2_

**Figure 13:**
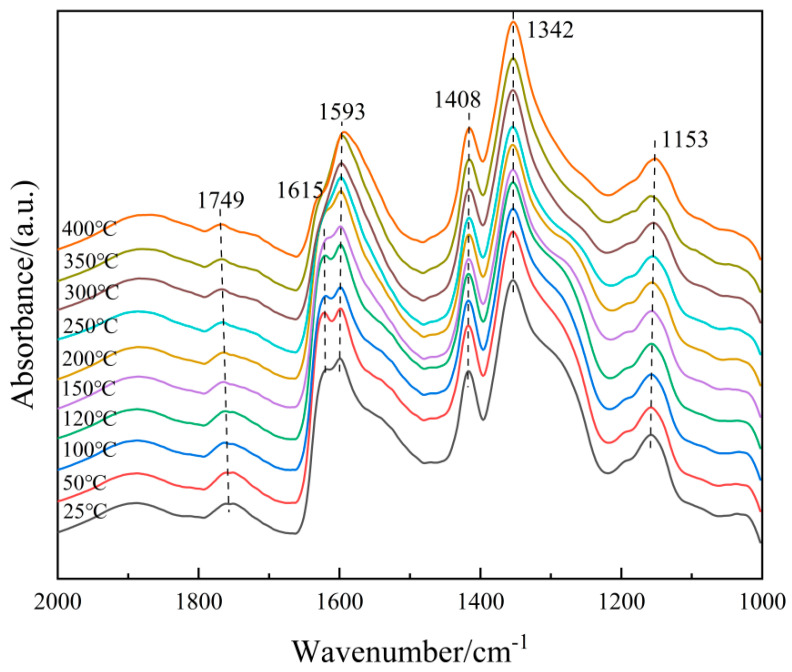
In situ DRIFTS spectra of NO + O_2_ adsorption on 6Fe_2_O_3_-6CeO_2_-40MnO_2_/TiO_2_ catalyst.

To understand the microscopic process of the NH_3_-SCR reaction on the 6Fe_2_O_3_-6CeO_2_-40MnO_2_/TiO_2_ catalyst, it is necessary to distinguish the adsorbent species on the surface of the catalyst. Therefore, an in situ co-adsorption experiment of NO + O_2_ was carried out on the 6Fe_2_O_3_-6CeO_2_-40MnO_2_/TiO_2_ catalyst, and the results are shown in Figure 13. The 6Fe_2_O_3_-6CeO_2_-40MnO_2_/TiO_2_ catalyst was exposed to a NO + O_2_ mixed gas (500 ppm NO + 5% O_2_, N_2_ balance) at room temperature, and multiple infrared vibration signal peaks appeared between 1000 and 2000 cm^−1^. Among them, the bands located at 1408 cm^−1^ and 1593 cm^−1^ can be attributed to bidentate nitrates; the band located at 1153 cm^−1^ is related to monodentate nitrates; the band located at 1342 cm^−1^ belongs to M-NO_2_ nitro compounds; the band located at 1615 cm^−1^ belongs to adsorbed NO_2_; and the band located at 1749 cm^−1^ belongs to the physical adsorption of NO_2_ [31,32]. As can be seen from Figure 11, as the temperature rises, the absorption peak intensity corresponding to monodentate nitrate (1153 cm^−1^) gradually decreases with increasing temperature, while the absorption peaks of bidentate nitrates (1408 cm^−1^ and 1593 cm^−1^) appear at lower temperatures and are not easily decomposed. This is due to the low thermal stability of the monodentate nitrate and a certain degree of desorption on the surface of the catalyst at high temperatures, which is consistent with the fact that bidentate nitrates are more thermally stable than monodentate nitrates. The peak intensity corresponding to the adsorbed NO_2_ (1615 cm^−1^) gradually decreases with increasing temperature, which is probably related to the effect of NO oxidation and the accelerated desorption of NO_2_ after the temperature increase.

(3) The microscopic reaction process of the catalyst

**Figure 14:**
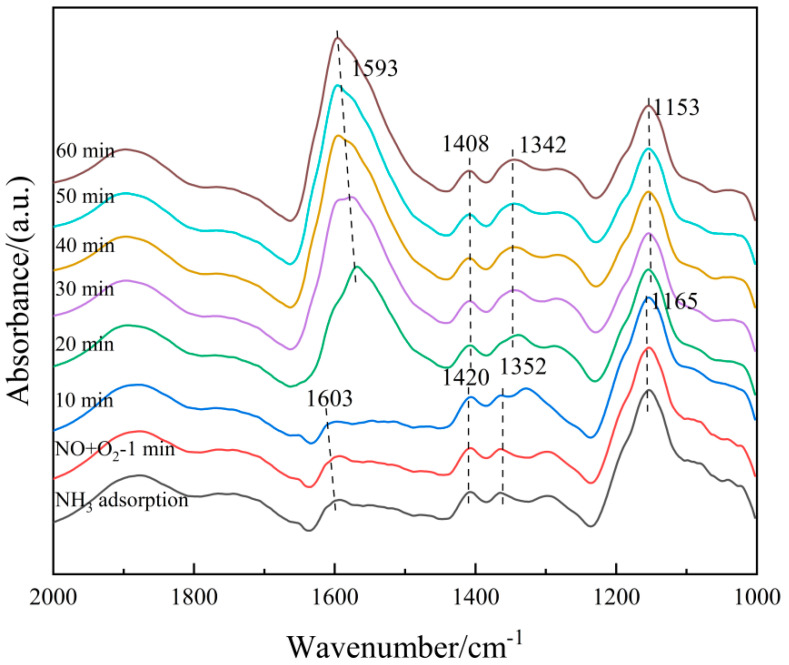
In situ DRIFTS spectra of NO + O_2_ adsorption on 6Fe_2_O_3_-6CeO_2_-40MnO_2_/TiO_2_ catalyst with pre-adsorption of NH_3_.

The 6Fe_2_O_3_-6CeO_2_-40MnO_2_/TiO_2_ catalyst pre-adsorbed with NH_3_ was subjected to in situ co-adsorption tests of NO + O_2_, in order to explore the microscopic process of the NH_3_-SCR reaction on the 6Fe_2_O_3_-6CeO_2_-40MnO_2_/TiO_2_ catalyst at 250 °C. The results are shown in Figure 14. At 250 °C, when NH_3_ adsorption is saturated, signals corresponding to Lewis acid sites (1165 cm^−1^, 1352 cm^−1^, and 1603 cm^−1^) and Brønsted acid sites (1420 cm^−1^) can be observed. Then, the NH_3_ gas supply was turned off, and a NO + O_2_ mixed gas (500 ppm NO + 5% O_2_, balanced with N_2_) was introduced. The following phenomena were observed over time: After 20 min of NO + O_2_ exposure, the signals related to NH_3_ adsorption completely disappeared, indicating that the NH_3_ species adsorbed on the catalyst surface had almost entirely participated in the reaction. Subsequently, absorption peaks corresponding to bidentate nitrates (1408 cm^−1^), monodentate nitrates (1153 cm^−1^), M-NO_2_ nitro compounds (1342 cm^−1^), and adsorbed NO_2_ (1602 cm^−1^) appeared. The intensity of the bidentate nitrate peak at 1408 cm^−1^ further increased with prolonged reaction time, likely due to the accumulation of bidentate nitrate species after the surface-adsorbed NH_3_ species were fully consumed. These results indicate that the 6Fe_2_O_3_-6CeO_2_-40MnO_2_/TiO_2_ catalyst can adsorb NO + O_2_ and convert it into various nitrate species. These nitrate species then react with the NH_3_ species previously adsorbed on Lewis and Brønsted acid sites to complete the denitration reaction cycle. The NH_3_-SCR reaction on the catalyst surface follows the L-H mechanism, as shown in Equations (3)–(11) [33].

(3) $$NH_{3}\left(g\right)\overset{Lewis\;acid\;sites}{\to }NH_{3}\left(ad\right)$$

(4) $$NO\left(g\right)\to NO\left(ad\right)$$

(5) $$\equiv M^{n+}=O+NO\left(ad\right)\to \equiv M^{\left(n-1\right)+}\text{-}O\text{-}NO$$

(6) $$M^{\left(n-1\right)+}\text{-}O\text{-}NO+NH_{3}\left(ad\right)\to \equiv M^{\left(n-1\right)+}\text{-}O\text{-}NO\text{-}NH_{3}\to \equiv M^{\left(n-1\right)+}\text{-}OH+N_{2}\left(g\right)+H_{2}O$$

(7) $$\equiv M^{\left(n-1\right)+}\text{-}OH+\frac{1}{4}O_{2}\to \equiv M^{n+}=O+\frac{1}{2}H_{2}O$$

(8) $$NH_{3}\left(g\right)\overset{Brønsted\;acid\;sites}{\to }{NH_{4}}^{+}\left(ad\right)$$

(9) $$O_{2}\left(g\right)\to 2O\left(ad\right)$$

(10) $$NO\left(g\right)+O\left(ad\right)\to NO_{2}\left(ad\right)$$

(11) $${NH_{4}}^{+}\left(ad\right)+e^{-}+NO_{2}\left(ad\right)\to NH_{4}NO_{2}\left(ad\right)\to N_{2}+2H_{2}O$$

**Figure 15:**
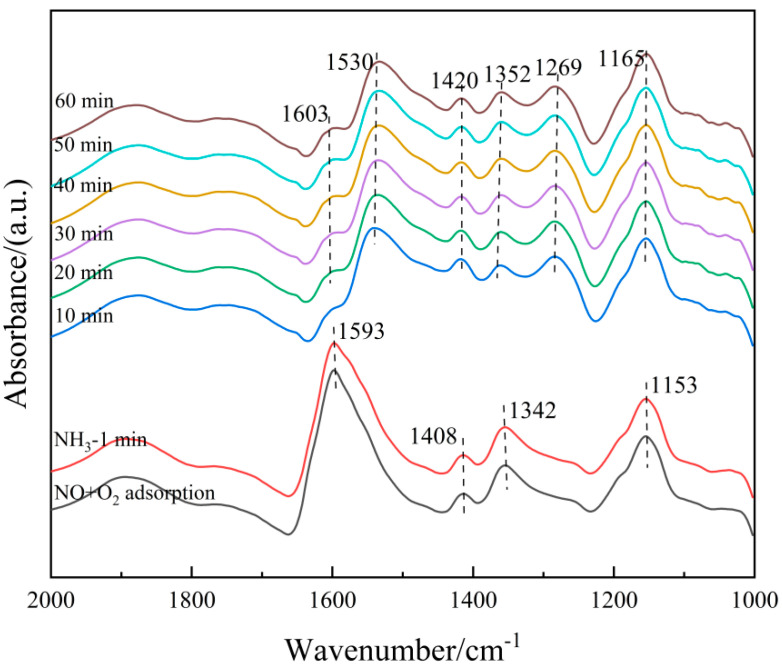
In situ DRIFTS spectra of NH_3_ adsorption on 6Fe_2_O_3_-6CeO_2_-40MnO_2_/TiO_2_ catalyst with pre-adsorption of NO + O_2_.

On the other hand, the 6Fe_2_O_3_-6CeO_2_-40MnO_2_/TiO_2_ catalyst pre-adsorbed with NO + O_2_ was subjected to in situ NH_3_ adsorption at 250 °C, as shown in Figure 15. When the 6Fe_2_O_3_-6CeO_2_-40MnO_2_/TiO_2_ catalyst adsorbs NO + O_2_ at 250 °C and reaches saturation, the absorption peaks of bidentate nitrate (1408 cm^−1^), monodentate nitrate (1153 cm^−1^), bridged nitrate (1269 cm^−1^), M-NO_2_ nitro compound (1342 cm^−1^), and adsorbed NO_2_ (1602 cm^−1^) can be detected. Then, the NO + O_2_ mixed gas is turned off and NH_3_ gas is introduced. Over time, the following phenomena occur: When NH_3_ gas is introduced for 10 min, the signals related to the adsorption of NO + O_2_ disappear, indicating that the nitrate species adsorbed on the surface of the catalyst have fully participated in the reaction. After 10 min, the Lewis acid sites (1165 cm^−1^, 1352 cm^−1^, and 1603 cm^−1^) and Brønsted acid sites (1269 cm^−1^, 1420 cm^−1^, and 1530 cm^−1^) adsorb NH_3_ species, forming corresponding absorption peaks. The intensity of each absorption peak does not change significantly with the extension of reaction time, indicating that the reaction is complete at this point. The above results show that the NO_x_ species adsorbed on the surface of the 6Fe_2_O_3_-6CeO_2_-40MnO_2_/TiO_2_ catalyst can react with gaseous NH_3_ molecules, indicating that the NH_3_-SCR reaction on the catalyst surface also follows the E-R mechanism. Combining Figure 14 and Figure 15, it can be concluded that the NH_3_-SCR reaction on the surface of the 6Fe_2_O_3_-6CeO_2_-40MnO_2_/TiO_2_ catalyst follows both the L-H and E-R mechanisms, with the L-H mechanism being dominant.

## 3. Materials and Methods

### 3.1. Catalyst Preparation

**Figure 16:**
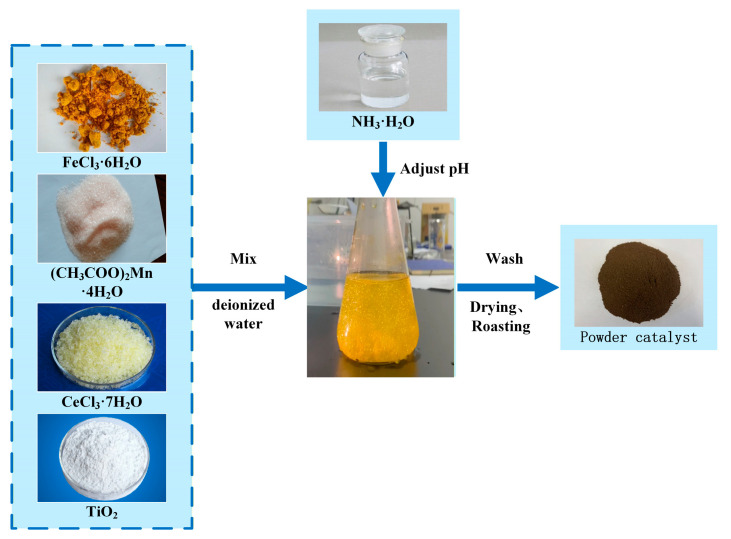
xFe_2_O_3_-6CeO_2_-40MnO_2_/TiO_2_ catalyst preparation process.

The Fe_2_O_3_-CeO_2_-MnO_2_/TiO_2_ catalyst was prepared by the co-precipitation method. The preparation steps are as follows: Manganese acetate tetrahydrate ((CH_3_COO)_2_Mn·4H_2_O, China National Pharmaceutical Group Chemical Reagent Co., Ltd., Shenyang, China), cerium chloride heptahydrate (CeCl_3_·7H_2_O, China National Pharmaceutical Group Chemical Reagent Co., Ltd., Shenyang, China), iron chloride hexahydrate (FeCl_3_·6H_2_O, China National Pharmaceutical Group Chemical Reagent Co., Ltd., Shenyang, China), and anatase (TiO_2_, China National Pharmaceutical Group Chemical Reagent Co., Ltd., Shenyang, China) were added to a beaker containing deionized water in a specific weight ratio. The mixture was continuously stirred for 0.5 h, and ammonia was then added dropwise to the catalyst solution until the pH reached 9~10. After that, the mixture was stirred for an additional 3 h and filtered, and deionized water was added to the filter cake. The mixture was stirred and washed for 0.5 h, and then filtered again. These operations were repeated three times. The resulting filter cake was dried at 75 °C for 24 h. Finally, the dried filter cake was placed in a muffle furnace and roasted at 400 °C for 4 h to obtain the xFe_2_O_3_-6CeO_2_-40MnO_2_/TiO_2_ catalyst. The x-value represents the mass percentage of Fe_2_O_3_ in the entire catalyst (×100), and the values used were 0, 2, 4, 6 and 8. The actual x-value was determined by ICP and rounded to the nearest integer. The preparation process is shown in Figure 16.

### 3.2. Catalyst Characterization

X-ray diffraction (XRD) analysis was conducted using an X-ray diffractometer, model JSM-7800F (Tokyo, Japan), manufactured by the Japanese Electronics Company. The test conditions included a Cu target, Kα radiation, an analysis voltage of 40 kV, an analysis current of 40 mA, a scanning range of 5° to 90°, and a scanning rate of 2° per minute.

The N_2_ adsorption–desorption curve was measured using the ASAP-2020 (Mike Instrument Company, Washington, DC, USA) physical adsorption instrument from Micromeritics Company in the United States. Prior to the test, the sample was vacuum-treated at 400 °C for 4 h. The Brunauer–Emmett–Teller (BET) method was used to calculate the specific surface area of the sample, and the Barrett–Joyner–Halenda (BJH) method was employed to determine the pore size distribution.

For scanning electron microscope (SEM) analysis, a Hitachi SU8020 (Hitachi, Tokyo, Japan) ultra-high-resolution field emission scanning electron microscope was used, with a magnification range from 300,000 to 800,000 times. The X-ray spectrometer attached to the SEM has an elemental analysis range from B5 to U92 and an energy resolution of 130 eV.

A JEM-2100F transmission electron microscope (Nippon Electronics Corporation, Osaka, Japan) was used, which has a point resolution of 0.23 nm, a magnification range of 50 to 1,000,000, and an acceleration voltage of 200 kV. With this instrument, we observed the particle size and microstructure of the catalyst and conducted nanoscale analysis of the catalyst through high-resolution lattice fringe images.

X-ray photoelectron spectroscopy (XPS) was carried out using the Thermo Fisher Scientific Escalab 250Xi X-ray Photoelectron Spectrometer (Thermo Fisher Scientific, New York, NY, USA). The excitation source was Al Kα (1486.6 eV), and the acceleration voltage was 150 W. The binding energy of C 1s (284.8 eV) was used as the internal standard. Peak fitting was performed using XPS Peak 4.1 software.

Temperature-Programmed Reduction (TPR) was analyzed using a Micromeritics Autochem II 2920 chemical adsorption instrument (Micromeritics, New York, NY, USA). The test conditions are as follows: Take about 70 mg of sample and place it in a quartz tube; heat it up to 300 °C for drying and pretreatment; purify with He gas for 1 h, and then cool it to room temperature; pass a 10% H_2_/Ar mixture through it for 30 min until the baseline is stable; finally, heat it up to 800 °C in a 10% H_2_/Ar atmosphere for TPR. The gas flow rate is 50 mL/min, and the heating rate is 10 °C/min. The detector used is a Thermal Conductivity Detector (TCD), which detects the consumption of the reducing gas.

Temperature-programmed desorption (TPD) tests were carried out on an AutoChem II 2920 Chemisorption Analyzer (Micromeritics, New York, NY, USA). The specific conditions were as follows: approximately 70 mg of the sample was weighed and placed into a quartz tube; the sample was heated to 300 °C for drying and pretreatment under a flowing He gas atmosphere. After 1 h, cool it to room temperature. Subsequently, a 10% NH_3_/He gas mixture was introduced for 1 h to achieve saturation; the gas was switched to He, and the sample was purged for 1 h to remove physically adsorbed NH_3_. Finally, the sample was heated to 800 °C at a rate of 10 °C/min under a He gas flow (50 mL/min), and the desorbed gas was detected using a TCD.

In situ Diffuse Reflectance Infrared Fourier Transform Spectroscopy (In situ DRIFTS) was performed using a Thermo Scientific Nicolet IS50 Fourier Transform Infrared (FTIR) spectrometer (Thermo Scientific, Waltham, MA, USA). The measurement procedure was as follows: approximately 15 mg of sample was placed flat in a small crucible and fixed inside a high-temperature in situ cell. The sample was pretreated with N_2_ at 400 °C for 1 h, then cooled to room temperature. Background spectra (32 scans, 4 cm^−1^ resolution) were collected periodically as references. Subsequently, the corresponding reaction gas (1000 ppm NH_3_, 1000 ppm NO, and 5% O_2_ by volume) was introduced for in situ infrared adsorption studies. For NH_3_ or NO + O_2_ adsorption, the gas was introduced at 200 °C, and data were collected over time. For the experiment involving initial NH_3_ pre-adsorption followed by NO + O_2_ exposure, saturated NH_3_ adsorption was first conducted at 200 °C, after which the NO + O_2_ mixture was introduced, and data were collected over time. Similarly, for the experiment involving initial NO + O_2_ pre-adsorption followed by NH_3_ exposure, saturated NO + O_2_ adsorption was performed at 200 °C, followed by NH_3_ introduction, and data were collected over time. At the end, the background spectrum was subtracted to obtain the infrared spectra at each time point.

### 3.3. Measurement of Catalyst Activity

**Figure 17:**
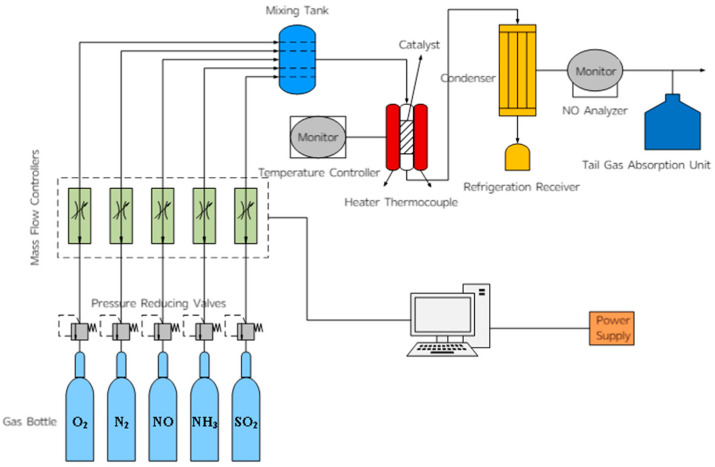
Schematic diagram of fixed-bed catalytic reactor.

The laboratory micro-reaction device was used as shown in Figure 17 to simulate the components of the flue gas. The test conditions were as follows: a 5 mL catalyst sample, a gas hourly space velocity (GHSV) of 36,000 h^−1^, and a simulated flue gas composition consisting of 600 ppm NO, 300 ppm O_2_, 600 ppm NH_3_, and 60 ppm SO_2_ (when required), with N_2_ as the balance gas. Five milliliters of the catalyst to be tested were placed in a fixed-bed reactor, the flow control system was turned on, and the gas parameters were set. The pipeline was purged with N_2_, and the air tightness of the system was checked after 5 min. Then the final temperature was set, the temperature control switch was turned on, other gases were introduced, and the NO concentration detected by the analyzer was recorded as the temperature changed. After the test was completed, the other gas switches were turned off, N_2_ continued to flow, and the pipeline was purged with N_2_ until the NO detection indicator dropped to zero (NH_3_ analyzer model: Taihe Lianchuang THA100 (Taihe Lianchuang, Beijing, China); The models of NO, N_2_O, and NO_2_ analyzers are Wuhan Sensuo Technology SS7200, Wuhan, China).

The equations for calculating the denitration efficiency and N_2_ selectivity of the catalyst are as follows:

(12) $$\mathrm{NO}\;Conversion=\frac{{\left[NO\right]}_{in}-{\left[NO\right]}_{out}}{{\left[NO\right]}_{in}}\times 100\%$$

(13) $$N_{2}\;Selectivity=\left[1-\frac{2\left[N_{2}O\right]}{{\left[NH_{3}\right]}_{in}-{\left[NH_{3}\right]}_{out}+{\left[NO\right]}_{in}-{\left[NO\right]}_{out}}\right]\times 100\%$$

where [*NO*]*_in_* represents the input concentration of NO, [*NO*]*_out_* denotes the output concentration of NO, [*NH*_3_]*_in_* signifies the input concentration of NH_3_, [*NH*_3_]*_out_* refers to the output concentration of NH_3_, and [*N*_2_*O*] is the output concentration of N_2_O.

## 4. Conclusions

In this study, based on the Ce-Mn/TiO_2_ catalyst, Fe_2_O_3_ was introduced for modification, and a new type of xFe_2_O_3_-6CeO_2_-40MnO_2_/TiO_2_ catalyst was prepared. Its denitration properties and reaction kinetics in the NH_3_ selective catalytic reduction in the NO (SCR) reaction were systematically investigated. The experimental results show that the 6Fe_2_O_3_-6CeO_2_-40MnO_2_/TiO_2_ catalyst modified with 6% Fe_2_O_3_ exhibits the best denitration performance. This catalyst has the lowest reaction activation energy (*Ea* = 56 kJ·mol^−1^), and its denitration efficiency exceeds 80% and 90% within the temperature ranges of 115~425 °C and 129~390 °C, respectively. The denitration efficiency reaches as high as 99.17% within the temperature range of 220–305 °C. Additionally, it exhibits good N_2_ selectivity and excellent resistance to SO_2_ and H_2_O. To conduct an in-depth analysis of the influence of Fe_2_O_3_ addition on catalyst performance, the xFe_2_O_3_-6CeO_2_-40MnO_2_/TiO_2_ catalyst was systematically studied using various characterization methods, such as XRD, BET, SEM, TEM, XPS, TPR, and TPD. The results show that after the addition of 6% Fe_2_O_3_, the TiO_2_ lattice contracts to some extent, enhancing its dispersion and significantly increasing the specific surface area of the catalyst. In addition, the content of chemically adsorbed oxygen on the catalyst surface and the proportion of low-cost metal ions such as Fe^2+^, Ce^3+^, and Mn^2+^ have increased substantially, providing more oxygen vacancies and active sites for the SCR reaction. Meanwhile, the number of strong acid sites on the catalyst surface has increased notably, and the redox capacity has been enhanced, which facilitates the NH_3_-SCR reaction. Further research has found that the introduction of Fe_2_O_3_ promotes the formation of strong acid sites, while CeO_2_, as the main active component, is more inclined to generate weak and medium-strong acid sites, thereby creating a favorable synergistic effect between the two. Through the analysis of in situ DRIFTS, it can be seen that the NH_3_-SCR reaction mechanism on the 6Fe_2_O_3_-6CeO_2_-40MnO_2_/TiO_2_ catalyst follows both the L-H and E-R mechanisms, with the L-H mechanism being the dominant reaction pathway.

## Figures and Tables

**Table 1:** Specific surface area and pore size parameters of xFe_2_O_3_-6CeO_2_-40MnO_2_/TiO_2_ catalysts.

Catalyst	Specific Surface (m^2^·g^−1^)	Pore Capacity (cm^3^·g^−1^)	Pore Size (nm)
6CeO_2_-40MnO_2_/TiO_2_	83	0.218	16.37
2Fe_2_O_3_-6CeO_2_-40MnO_2_/TiO_2_	84	0.252	15.19
4Fe_2_O_3_-6CeO_2_-40MnO_2_/TiO_2_	87	0.257	13.68
6Fe_2_O_3_-6CeO_2_-40MnO_2_/TiO_2_	88	0.268	12.22
8Fe_2_O_3_-6CeO_2_-40MnO_2_/TiO_2_	88	0.254	13.34

**Table 2:** Surface element content of xFe_2_O_3_-6CeO_2_-40MnO_2_/TiO_2_ catalysts (mass, %).

Catalyst	Fe	Ce	Mn	Ti	O
6CeO_2_-40MnO_2_/TiO_2_	0	5.36	11.86	13.05	69.73
2Fe_2_O_3_-6CeO_2_-40MnO_2_/TiO_2_	2.87	4.98	11.43	11.27	69.45
4Fe_2_O_3_-6CeO_2_-40MnO_2_/TiO_2_	4.03	4.47	10.94	10.68	69.88
6Fe_2_O_3_-6CeO_2_-40MnO_2_/TiO_2_	4.96	4.51	9.98	10.30	70.25
8Fe_2_O_3_-6CeO_2_-40MnO_2_/TiO_2_	5.78	4.23	8.55	10.46	70.98

**Table 3:** NH_3_ adsorption capacity of xFe_2_O_3_-6CeO_2_-40MnO_2_/TiO_2_ catalysts.

Catalyst	NH_3_ Adsorption at the Weak Acid Sites (μmol·g^−1^)	NH_3_ Adsorption at the Medium-Strong Acid Sites (μmol·g^−1^)	NH_3_ Adsorption at the Strong Acid Sites (μmol·g^−1^)
6CeO_2_-40MnO_2_/TiO_2_	185.32	303.22	868.57
2Fe_2_O_3_-6CeO_2_-40MnO_2_/TiO_2_	188.56	304.73	957.34
4Fe_2_O_3_-6CeO_2_-40MnO_2_/TiO_2_	189.85	311.94	1079.65
6Fe_2_O_3_-6CeO_2_-40MnO_2_/TiO_2_	187.15	318.66	1108.59
8Fe_2_O_3_-6CeO_2_-40MnO_2_/TiO_2_	182.77	321.65	1089.73

**Table 4:** H_2_ consumption of xFe_2_O_3_-6CeO_2_-40MnO_2_/TiO_2_ catalysts.

Catalyst	Fe^3+^ → Fe^2+^ H_2_ Consumption (mmol·g^−1^)	Mn^4+^ → Mn^3+^ H_2_ Consumption (mmol·g^−1^)	Ce^4+^ → Ce^3+^ H_2_ Consumption (mmol·g^−1^)	Total Consumption of H_2_ (mmol·g^−1^)
6CeO_2_-40MnO_2_/TiO_2_	3.78	5.45	0.63	9.86
2Fe_2_O_3_-6CeO_2_-40MnO_2_/TiO_2_	3.50	5.21	0.51	9.22
4Fe_2_O_3_-6CeO_2_-40MnO_2_/TiO_2_	3.76	5.68	0.98	10.42
6Fe_2_O_3_-6CeO_2_-40MnO_2_/TiO_2_	4.18	5.73	1.34	11.25
8Fe_2_O_3_-6CeO_2_-40MnO_2_/TiO_2_	4.02	5.31	1.03	10.36

## Data Availability

The raw data supporting the conclusions of this article will be made available by the authors on request.
